# Setting a baseline for global urban virome surveillance in sewage

**DOI:** 10.1038/s41598-020-69869-0

**Published:** 2020-08-13

**Authors:** David F. Nieuwenhuijse, Bas B. Oude Munnink, My V. T. Phan, the Global Sewage Surveillance project consortium, Rene S. Hendriksen, Artan Bego, Catherine Rees, Elizabeth Heather Neilson, Kris Coventry, Peter Collignon, Franz Allerberger, Teddie O. Rahube, Guilherme Oliveira, Ivan Ivanov, Thet Sopheak, Yith Vuthy, Christopher K. Yost, Djim-adjim Tabo, Sara Cuadros-Orellana, Changwen Ke, Huanying Zheng, Li Baisheng, Xiaoyang Jiao, Pilar Donado-Godoy, Kalpy Julien Coulibaly, Jasna Hrenovic, Matijana Jergović, Renáta Karpíšková, Bodil Elsborg, Mengistu Legesse, Tadesse Eguale, Annamari Heikinheimo, Jose Eduardo Villacis, Bakary Sanneh, Lile Malania, Andreas Nitsche, Annika Brinkmann, Courage Kosi Setsoafia Saba, Bela Kocsis, Norbert Solymosi, Thorunn R. Thorsteinsdottir, Abdulla Mohamed Hatha, Masoud Alebouyeh, Dearbhaile Morris, Louise O’Connor, Martin Cormican, Jacob Moran-Gilad, Antonio Battisti, Patricia Alba, Zeinegul Shakenova, Ciira Kiiyukia, Eric Ng’eno, Lul Raka, Aivars Bērziņš, Jeļena Avsejenko, Vadims Bartkevics, Christian Penny, Heraa Rajandas, Sivachandran Parimannan, Malcolm Vella Haber, Pushkar Pal, Heike Schmitt, Mark van Passel, Milou G.M. van de Schans, Tina Zuidema, Gert-Jan Jeunen, Neil Gemmell, Kayode Fashae, Astrid Louise Wester, Rune Holmstad, Rumina Hasan, Sadia Shakoor, Maria Luz Zamudio Rojas, Dariusz Wasyl, Golubinka Bosevska, Mihail Kochubovski, Cojocaru Radu, Amy Gassama†, Vladimir Radosavljevic, Moon Y.F. Tay, Rogelio Zuniga-Montanez, Stefan Wuertz, Dagmar Gavačová, Marija Trkov, Karen Keddy, Kerneels Esterhuyse, Marta Cerdà-Cuéllar, Sujatha Pathirage, D.G.Joakim Larsson, Leif Norrgren, Stefan Örn, Tanja Van der Heijden, Happiness Houka Kumburu, Ana Maria de RodaHusman, Berthe-Marie Njanpop-Lafourcade, Pawou Bidjada, Somtinda Christelle Nikiema-Pessinaba, Belkis Levent, John Scott Meschke, Nicola Koren Beck, Chinh Van Dang, Doan Minh Nguyen Tran, Nguyen Do Phuc, Geoffrey Kwenda, Patrick Munk, Shweta Venkatakrishnan, Frank M. Aarestrup, Matthew Cotten, Marion P. G. Koopmans

**Affiliations:** 1 Viroscience Department, Erasmus Medical Center, Rotterdam, The Netherlands; 2 National Food Institute, Technical University of Denmark, Lyngby, Denmark; 3 Institute of Public Health, Tirana, Albania; 4 Melbourne Water Corporation, Docklands, Australia; 5 University of Copenhagen, Frederiksberg C, Australia; 6 Applied Research, Docklands, Australia; 7 Canberra Hospital, Canberra, Australia; 8 Austrian Agency for Health and Food Safety (AGES), Vienna, Austria; 9 Botswana International University of Science and Technology, Palapye, Botswana; 10 Vale Institute of Technology, Sustainable Development, Belém, Brazil; 11 National Center of Infectious and Parasitic Diseases, Sofia, Bulgaria; 12 Institut Pasteur du Cambodge, Phnom Penh, Cambodia; 13 University of Regina, Regina, Canada; 14 University of N’Djamena, N’Djamena, Chad; 15 Centro de Biotecnología de los Recursos Naturales, Universidad Católica del Maule, Talca, Chile; 16 Guangdong Provincial Center for Disease Control and Prevention, Guangzhou, China; 17 Shantou University Medical College, Shantou, China; 18 Corporacion Colombiana de Investigacion Agropecuaria (AGROSAVIA), Mosquera, Colombia; 19 Institut Pasteur de Côte d’Ivoire, Abidjan, Côte d’Ivoire; 20 Faculty of Science, University of Zagreb, Zagreb, Croatia; 21 Andrija Stampar Teaching Institute of Public Health, Zagreb, Croatia; 22 Veterinary Research Institute, Brno, Czech Republic; 23 Renseanlæg Lynetten, København K, Denmark; 24 Addis Ababa University, Addis Ababa, Ethiopia; 25 University of Helsinki, Helsinki, Finland; 26 Instituto Nacional de Investigación en Salud Pública-INSPI (CRNRAM), Quito, Galápagos, Ecuador; 27 National Public Health Laboratories, Ministry of Health and Social Welfare, Kotu Layout, Kotu, Gambia; 28 National Center for Disease Control and Public Health, Tbilisi, Georgia; 29 Robert Koch Institute, Berlin, Germany; 30 University for Development Studies, Tamale, Ghana; 31 Semmelweis University, Institute of Medical Microbiology, Budapest, Hungary; 32 University of Veterinary Medicine, Budapest, Hungary; 33 Institute for Experimental Pathology, University of Iceland, Keldur, Reykjavík, Iceland; 34 Cochin University of Science and Technology, Cochin, India; 35 Pediatric Infections Research Center, Research Institute for Children’s Health, Shahid Beheshti University of Medical Sciences, Tehran, Iran; 36 National University of Ireland Galway, Galway, Ireland; 37 School of Public Health, Ben Gurion University of the Negev and Ministry of Health, Beer-Sheva, Israel; 38 Istituto Zooprofilattico Sperimentale del Lazio e della Toscana, Rome, Italy; 39 National Center of Expertise, Taldykorgan, Kazakhstan; 40 Mount Kenya University, Thika, Kenya; 41 Kenya Medical Research Institute, Nairobi, Kenya; 42 University of Prishtina “Hasan Prishtina” & National Institute of Public Health of Kosovo, Pristina, Kosovo; 43 Institute of Food Safety, Animal Health and Environment “BIOR”, Riga, Latvia; 44 Institute of Food Safety, Riga, Latvia; 45 Luxembourg Institute of Science and Technology, Belvaux, Luxembourg; 46 Centre of Excellence for Omics-Driven Computational Biodiscovery, Faculty of Applied Sciences, AIMST University, Kedah, Malaysia; 47 Environmental Health Directorate, St. Venera, Malta; 48 Agriculture and Forestry University, Kathmandu, Nepal; 49 National Institute for Public, Health and the Environment (RIVM), Bilthoven, Netherlands; 50 Wageningen Food Safety Research, Wageningen, Netherlands; 51 University of Otago, Dunedin, New Zealand; 52 University of Ibadan, Ibadan, Nigeria; 53 Norwegian Institute of Public Health, Oslo, Norway; 54 VEAS, Slemmestad, Norway; 55 Aga Khan University, Karachi, Pakistan; 56 National Institute of Health, Lima, Peru; 57 National Veterinary Research Institute, Puławy, Poland; 58 Institute of Public Health of the Republic of Macedonia, Skopje, Republic of Macedonia; 59 State Medical and Pharmaceutical University, Chişinău, Republic of Moldova; 60 Institut Pasteur de Dakar, Dakar, Sénégal; 61 Institute of Veterinary Medicine of Serbia, Belgrade, Serbia; 62 Nanyang Technological University Food Technology Centre (NAFTEC), Nanyang Technological University (NTU), Singapore, Singapore; 63 Nanyang Technological University, Singapore Centre for Environmental Life Sciences Engineering (SCELSE), Singapore, Singapore; 64 Public Health Authority of the Slovak Republic, Bratislava, Slovakia; 65 National Laboratory of Health, Environment and Food, Ljubljana, Slovenia; 66 University of the Witwatersrand, Johannesburg, South Africa; 67 Daspoort Waste Water Treatment Works, Pretoria, South Africa; 68 IRTA, Centre de Recerca en Sanitat Animal (CReSA, IRTA-UAB), Bellaterra, Spain; 69 Medical Research Institute, Colombo, Sri Lanka; 70 The Sahlgrenska Academy at the University of Gothenburg, Gothenburg, Sweden; 71 Swedish University of Agricultural Sciences, Uppsala, Sweden; 72 Ara region bern ag, Herrenschwanden, Switzerland; 73 Kilimanjaro Clinical Research Institute, Moshi, Tanzania; 74 Centre for Infectious Disease Control, Bilthoven, the Netherlands; 75 Agence de Médecine Préventive, Dapaong, Togo; 76 National Institute of Hygiene, Lome, Togo; 77 Division of Integrated Surveillance of Health Emergencies and Response, Lomé, Togo; 78 Public Health Institution of Turkey, Ankara, Turkey; 79 University of Washington, Seattle, USA; 80 Institute of Public Health in Ho Chi Minh City, Ho Chi Minh, Viet Nam; 81 University of Zambia, Lusaka, Zambia

**Keywords:** Metagenomics, Public health

## Abstract

The rapid development of megacities, and their growing connectedness across the world is becoming a distinct driver for emerging disease outbreaks. Early detection of unusual disease emergence and spread should therefore include such cities as part of risk-based surveillance. A catch-all metagenomic sequencing approach of urban sewage could potentially provide an unbiased insight into the dynamics of viral pathogens circulating in a community irrespective of access to care, a potential which already has been proven for the surveillance of poliovirus. Here, we present a detailed characterization of sewage viromes from a snapshot of 81 high density urban areas across the globe, including in-depth assessment of potential biases, as a proof of concept for catch-all viral pathogen surveillance. We show the ability to detect a wide range of viruses and geographical and seasonal differences for specific viral groups. Our findings offer a cross-sectional baseline for further research in viral surveillance from urban sewage samples and place previous studies in a global perspective.

## Introduction

The increasing connectivity of the modern world, changing demographics, and climate change increase the potential for novel and known viral pathogens to emerge and rapidly spread in new and unexpected areas, as could be seen during the emergence and global threat of Ebola virus in recent outbreaks^1^. Early detection or ruling out of high impact (emerging) infections as causes of disease is a hallmark of preparedness, but research in response to recent outbreaks of Ebola, Zika and yellow fever has shown that these pathogens circulated for extended periods of time before being recognized, leading to costly delays in public health response^2–5^. One of the key challenges is how to prioritize local investments in detection capacity, given the diversity of emerging diseases, the unpredictable nature of outbreaks, and the limited resources available for outbreak preparedness. Understandably, surveillance of infectious diseases mainly targets common conditions and is scaled up in response to the emergence of pathogens and in particular disease outbreaks, rather than the costlier approach of broad range testing for any relevant infectious disease. The changing dynamics of infectious diseases related to global change, however, require rethinking of this model for public health preparedness, as incidence-based surveillance provides a fragmented and limited scope of which pathogens are circulating in the general population, particularly in low resource settings where access to healthcare and laboratory diagnostics is restricted^6,7^. Therefore, in its reorganization in response to the West African Ebola outbreak, the World Health Organization has launched the term “Disease X” to call for novel ideas for preparedness to unpredictable disease outbreaks^8^. Thus, there is a need for novel approaches to viral surveillance providing a broader and less biased insight into the circulation of viral pathogens to supplement the more targeted surveillance. Genomic epidemiology using real-time pathogen sequencing has become part of the routine toolbox for outbreak tracking once the cause of the outbreak is known^9,10^. In addition, metagenomic sequencing has been put forward as a potential catch-all surveillance tool, but the step from research to routine implementation is extremely challenging^11,12^, and thus, careful validation is needed to avoid overpromise and wasting of resources.

Here, we set out to explore the potential use of metagenomic sequencing of urban sewage as an add-on strategy for global disease preparedness. One key driver of emergence is the amplification of rare zoonotic and vector-borne diseases in densely populated regions where infrastructure needs are outpaced by rapid urban developments. This leads to the formation of slums, favorable conditions for viral disease vectors, disparity in access to clean water, sanitation and healthcare, and an increase in close human-animal interaction due to deforestation^13,14^. The advantage of using sewage-based surveillance is that it represents the entire population of the catchment area, sample collection is straightforward, and the anonymization by default makes it less challenging to use than patient-based surveillance regarding privacy laws. Using sewage to detect viruses with low case fatality rate but overall high population level impact has been tested successfully to monitor the progress of the global polio-elimination program, particularly in regions where non-replicating polio-virus vaccines are used^15,16^. The huge potential of environmental surveillance was illustrated when a silent epidemic of wild-poliovirus type 1 in Israel was detected, which led to a mop-up vaccination campaign and resolution of the epidemic, without a single case of paralytic poliomyelitis^17^. In addition, small-scale studies have already shown the potential for using metagenomic sequencing of sewage extracts for the detection of a range of virus families^18–20^ (Table 1 in Appendix). While these studies have largely focused on viruses with a replication phase in the gastro-intestinal tract, the fecal and/or urinary shedding of, for instance, measles virus, yellow fever virus, Zika virus, West Nile virus, Ebola virus, SARS coronavirus, and MERS coronavirus suggests the potential utility of sewage testing to capture circulation of these pathogens as well^21–25^. Moreover, metagenomic sequencing has the potential to detect any viral genomic material in the sample, without targeting a specific viral pathogen or limiting for only known viral pathogens. In this study, we pilot the use of metagenomics to describe a comparative snapshot of the virome from sewage samples of high-density urban areas across all continents. We provide a critical appraisal of technical and analytical biases and discuss the potential utility for human and animal disease monitoring and surveillance, as well as the additional steps needed to go towards routine implementation.

## Results

### Data quality evaluation

**Figure 1:**
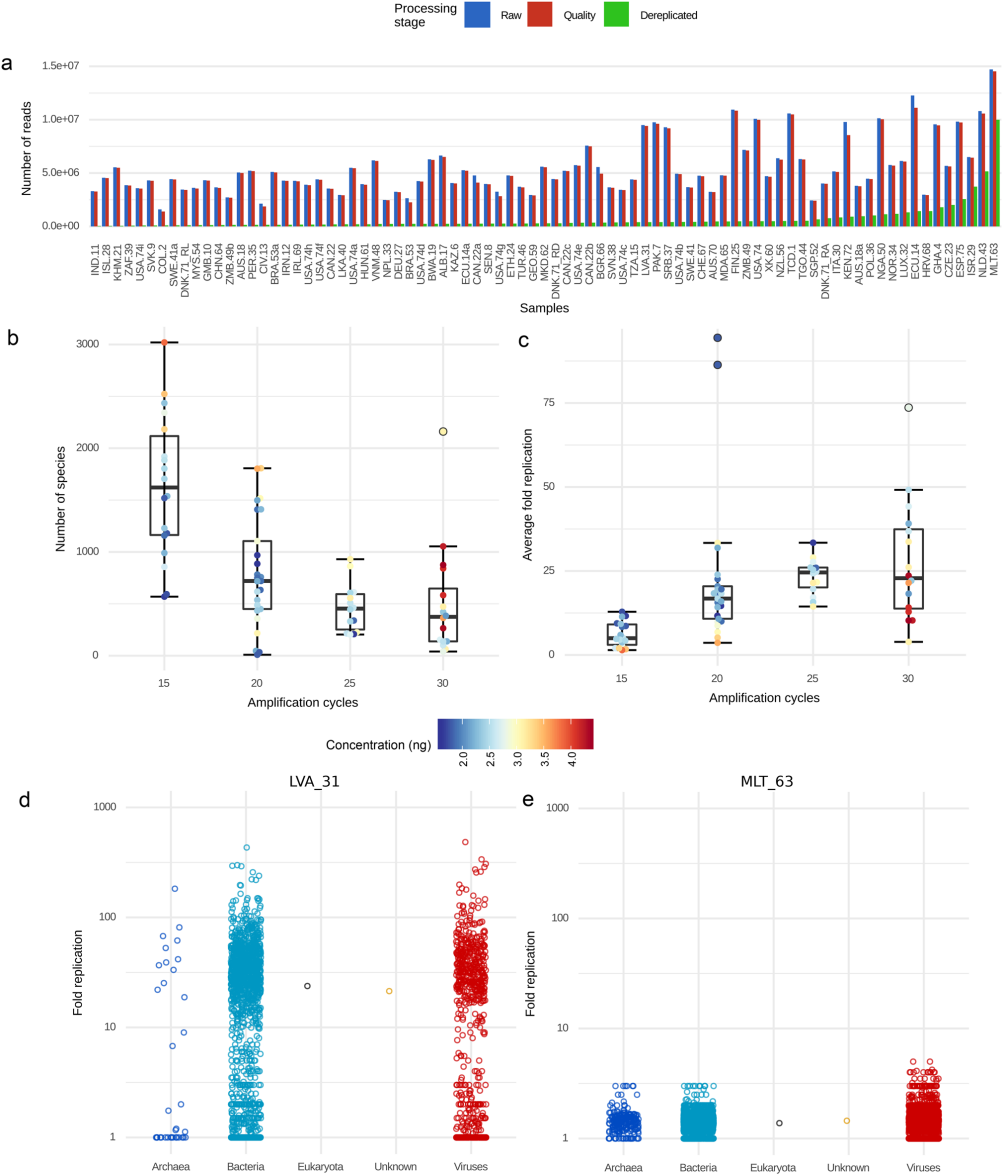
Effect of read preprocessing on data interpretation. (**a**) Number of reads before preprocessing (blue bars) after quality control (red bars) and read dereplication (green bars). The x axis shows sample identifiers ordered by number of dereplicated reads. (**b**, **c**) Effect of number of PCR replication cycles on library concentration (color), species diversity (**b**) and read replication rate (**c**). (**d**, **e**) Fold replication of raw reads by species level annotation (points). X axis separates superkingdom or “Unknown” annotations. (**d**) shows sample LVA_31 with a high replication rate and panel **e** shows sample MLT_63 with a low replication rate.

Urban sewage samples and associated metadata (Supp. File 1) were obtained from 62 countries across all continents between January and April 2016 from the influent of wastewater treatment plants prior to treatment or from open sewage systems in low- and middle-income countries. All samples were previously processed for the detection of bacterial antimicrobial resistance genes using DNA metagenomics^26^. Here we focus solely on viral DNA and RNA metagenomics (methods) and the analysis of the viral data. Sewage samples are highly variable in terms of composition and DNA abundance and therefore potential biases that might impact the final read abundance and diversity of the sewage virome were evaluated. Initially, an extensive evaluation of the technical factors that may impact the resulting data to gain a deeper understanding of potential pitfalls was performed. First, read abundance was evaluated as a proxy for viral abundance. Sequencing protocols for virome analysis in sewage typically require an amplification step to provide enough DNA input for sequencing, which can result in artificial duplication of sequence reads and thereby impact the quantitative interpretation of the data substantially (Fig. 1a). Indeed, the observed viral species richness was negatively correlated with the number of amplification cycles needed to obtain enough DNA as input for sequencing (Fig. 1b), while the average fold replication of a read was positively correlated (Fig. 1c). The impact of dereplication on the individual species level read counts varied greatly within a sample. Especially in samples with a low number of reads after dereplication (Fig. 1d) the decrease in read counts for a species ranges from 600 to fivefold . These differences have a profound effect on the species distribution within the sample, and thus the interpretation thereof. The effect of dereplication is much less variable between species in samples with a high number of reads after dereplication (Fig. 1e). Therefore, the optimal use of virome sequencing depends on the initial abundance of viral sequences in the sample and extra amplification may only increase the coverage of the same viruses, but does not increase the richness of the virome, which needs to be carefully considered when designing and interpreting sewage metagenomics studies.

Besides the influence of read replication on read abundance, the richness of the virome can be impacted by the presence of non-viral sequences. Typically, the metagenomic data contain a large fraction of unknown reads, and, despite the virus specific sample preparation, non-viral reads, including archaeal, bacterial, and eukaryote DNA.

**Figure 2:**
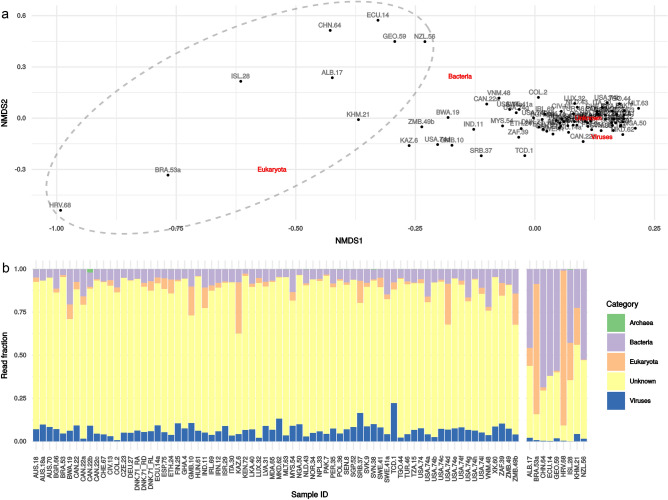
Effect of non-viral background read abundances on viral read abundance and the chosen outlier samples in the sewage metagenome data. (**a**) A multidimensional scaling of Bray–Curtis dissimilarity between samples based on the normalized read counts of bacterial, archaeal, eukaryote (human), and viral content. “Unknown” indicates reads that could not be assigned any annotation. The red labels indicate the effect of the different annotations on the position of a sample in the plot. Gray circle indicates the samples that were manually assigned to be outliers. (**b**) A scaled bar chart of relative read abundance showing the outliers in a separate facet to the right.

While the overall proportion of reads for the different domains was comparable in most samples, multidimensional scaling of the non-viral read counts showed that some samples were very divergent from the central cluster and were manually marked as outliers (Fig. 2a, dashed line). Viral read abundance was low in these outlier samples (Fig. 2b, right panel). There was no significant correlation between the concentration of human or bacterial read fractions with any of the measured sample characteristics, such as pH, conductivity, and type of sewer system.

### Exploration of the sewage virome

**Figure 3:**
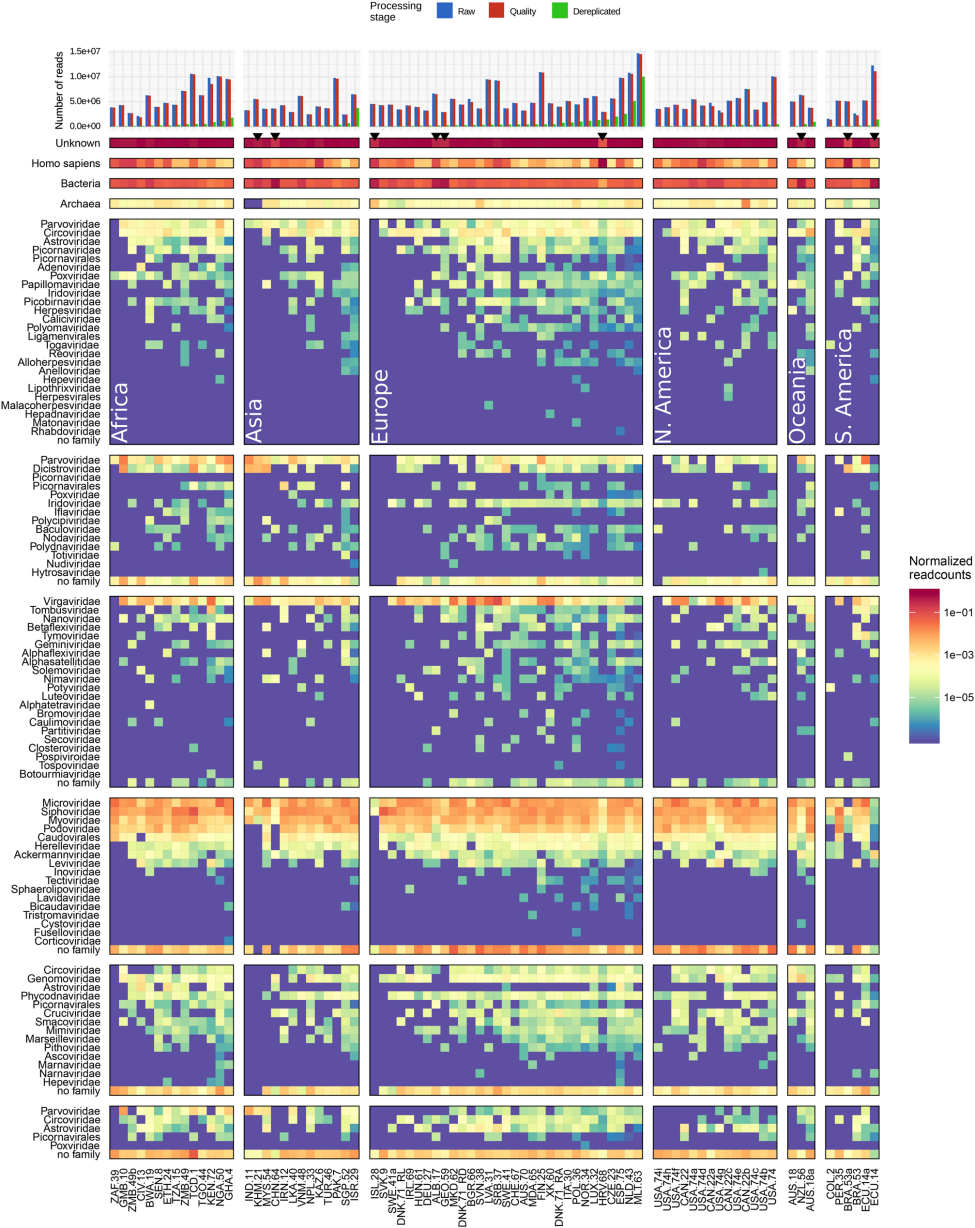
Heatmap of the viral diversity at viral family level (when available) and non-viral fraction. The read abundance after quality control and dereplication is shown ordered by total read abundance after preprocessing and facetted by continent. The heatmap follows the same ordering. Color gradient represents log-transformed relative abundance of reads belonging to the taxonomic groups indicated. The top four rows of the heatmap show read abundances of non-viral annotations, the other rows show read abundance by viral family, or “no family” if only genus or species level annotation was available. Vertical facets represent subdivision of the viral families based on their inferred host. Black arrows indicate outlier samples based on an overabundance of background sequences.

Based on the data quality assessment, we analyzed viral diversity in the samples after dereplication and following annotation by both Kajiu and Centrifuge as described. Between 0.09% and 22% of the reads could be annotated as viral (median of 6%), with high abundances of bacteriophages, plant- and insect viruses (Fig. 3). Most abundant were bacteriophages, representing on average 77% (ranging from 9 to 94%) of the annotated viral reads in the sewage. In particular *Microviridae* (median of 18%, range 0.5–51% of reads), *Siphoviridae* (median of 17%, range 0.22–67% of reads), *Myoviridae* (median of 9%, range 0.08–41% of reads), and *Podoviridae* (median of 4%, range 0.02–25% of reads), were highly abundant. These bacteriophage families could be found around the globe without obvious regional differences when using read annotations at this taxonomic level. Although specific bacteriophages have been studied extensively as potential indicators of human fecal pollution, bacteriophage taxonomy is relatively poorly defined, making accurate classification challenging at genus and species level^27,28^. Hence, geographical patterns at a more fine-grained level of annotation may be lost in our analysis. Moreover, interpretation of patterns of bacteriophage abundance could be obscured by the fact that bacteriophages can encounter bacterial hosts in the sewage in which they can multiply. As described elsewhere, the analysis of the bacterial resistomes of the same samples showed clear segregation of sequences from Africa and Asia versus those from Europe and the US^26^. A more detailed analysis is needed to assess if there is a relation between specific bacteriophages and the resistomes, as environmental viromes have been shown to be a potential reservoir for antimicrobial resistance genes^29^.

### Global patterns of viruses related to vegetable consumption and to insects detected in urban sewage

**Figure 4:**
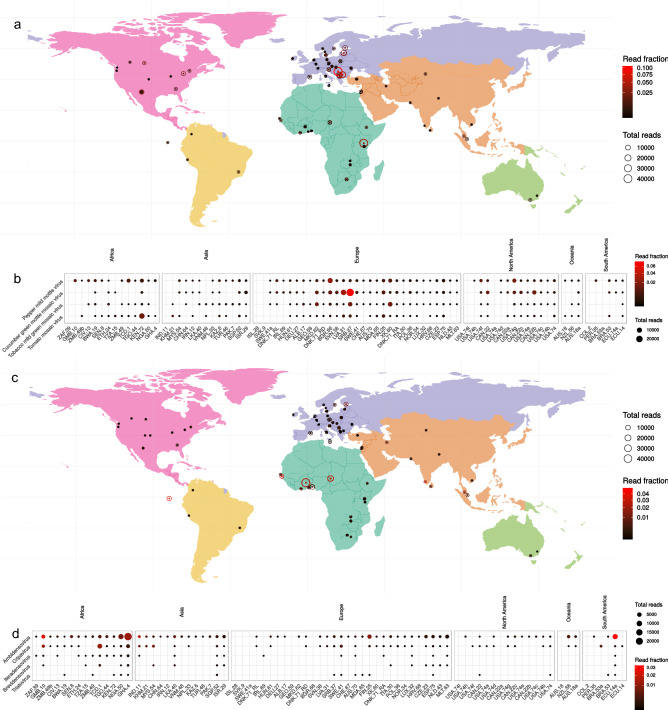
Overview of the global distribution and abundance of plant viruses and insect viruses in urban sewage (**a**) Global distribution of all plant viruses (**b**) The four most abundant plant virus species and their global spread. (**c**) Global distribution of all insect related viruses. (**d**) Top 5 most abundant insect virus genera. Datapoints represent absolute read numbers and read fraction by varying size and color respectively. Viral species are ordered by summed read abundance across samples and samples are ordered by total read abundance from left to right. Facets represent continent of sample origin.

The second largest fraction of the virome (0.02–69%, median 3.4%) consisted of plant-related viruses. On average, more than 84% of these reads belonged to the *Virgaviridae* family. Especially viral species related to infections of cucumber, tomato, tobacco and pepper plants could be detected in sewage, as indicated by species level taxonomy (Fig. 4b). Apart from a sample from Kenya, the abundance of vegetable-consumption-related viruses was higher in samples from Europe and North America compared to samples from the rest of the world (Fig. 4a) (Welch’s t-test, p-value = 0.06). The global presence and high abundance of plant viruses has led to the proposal that they may be good indicators for human fecal contamination alike specific bacteriophage populations^30^. However, this remains to be validated given the geographic variation observed in our dataset, which could reflect differences in diet and/or agricultural practices in these countries.

A median of 1.4% (ranging 0.1–74%) of the sewage virome consisted of viruses associated with insects, comprising mainly species from the genera *Ambidensovirus, Cripavirus,* and *Brevidensovirus* (Fig. 4d), known to infect a range of crickets, cockroaches, fruit flies, and mosquitos^31^. In the global distribution there was an increased abundance of insect viruses in samples from around the equator, mainly in samples from Africa (Fig. 4c) (Welch's t-test, p-value = 0.0004). One exception was the sample from Finland, which had a high abundance of insect virus reads (13.7%) in comparison with samples from other European countries (1.5%). Several reads were found to be annotated as “Aedes albopictus densovirus 2”, “Aedes aegypti Thai densovirus”, and “Anopheles gambiae densonucleosis virus”. There is some evidence that these densoviruses may be associated with *Aedes aegypti*, *Aedes albopictus* and *Culex* mosquitos^32,33^. Current data are not sufficient to meet the requirements for sewage surveillance, but these findings show the potential to track mosquitos by looking for mosquito specific viruses.

### Detection of vertebrate viruses and investigation of known human pathogens

**Figure 5:**
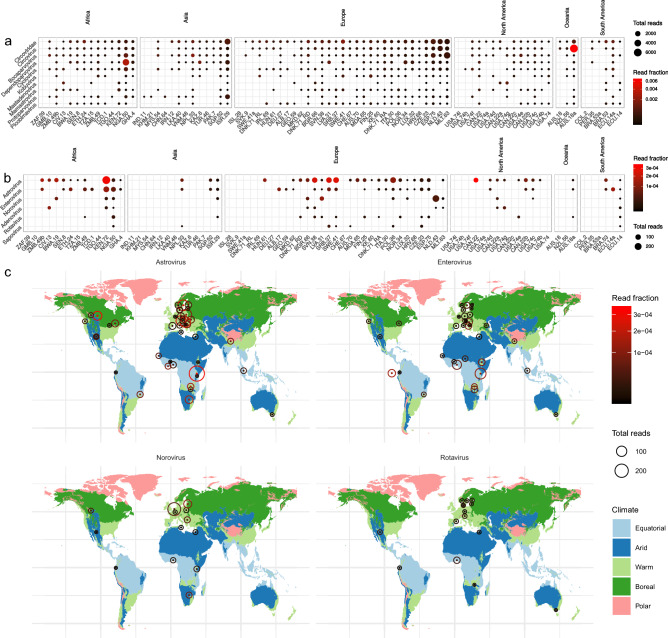
Overview of the most abundant vertebrate viruses and specific human viruses and their distribution worldwide in urban sewage. (**a**) Distribution of the top ten most abundant vertebrate viral families. (**b**) Relative abundance of viruses encountered in clinical surveillance (**c**) World maps showing distribution of viruses encountered in clinical surveillance. Coloring of the maps delineates differences in climate by geographical location. Datapoints represent absolute read numbers and read fraction by varying size and color respectively. Viral families are ordered by summed read abundance across samples and samples are ordered by total read abundance from left to right. Facets represent continent of sample origin.

About 1.7% (ranging 0.01–11%) of the virome consisted of vertebrate viruses. Most abundant were small ssDNA viruses from the families *Circoviridae* and *Parvoviridae,* and members of the *Picornaviridae, Astroviridae* and *Adenoviridae* families (Fig. 5a). Vertebrate viruses were detected widely across the samples, but did not show distinct geographical patterns of abundance. Circoviruses were especially highly abundant across most sewage samples and, as novel variants of circoviruses have been associated with several diseases in pigs^34^. Further longitudinal sewage surveillance could potentially be used to detect epidemiological patterns of emerging circovirus variants.

A selection of viral taxa was analyzed containing human pathogenic viruses from the *Astro*-, *Entero*- *Noro*-, *Sapo*-, *Adeno*- and *Rotaviridae* families that are known to be abundant across the world as causes of diarrheal disease (Fig. 5b). Most abundant and widespread were the astroviruses. Enteroviruses were present to a lesser extent but could be detected in sewage samples from across the globe as well. Members of the noro-, sapo-, adeno-, and rotaviruses were only sporadically detected. Further investigation of samples with high human astrovirus content showed mostly evidence of the classic Human Astrovirus 1, 2 and 4 that are common causes of diarrheal disease, and sporadic detection of other clades such as Human Astrovirus MLB and Human Astrovirus VA for which less is known regarding clinical impact^35^. Mapping of human enterovirus reads resulted in 102 small contiguous sequences which were typed using the enterovirus typing tool^36^. Mainly Enterovirus C (46%) and B (9%) were detected. Further subtyping of for instance poliovirus was not possible because of a lack of coverage of the standardized genotyping region VP1. The same mapping was done for norovirus, resulting in 13 contigs of 84 to 962 nucleotides in length. Most norovirus sequences were typed as either GII, with capsid type 6, 10 and 17, and GIV, all viruses that are commonly found in outbreak based surveillance^37^. Sapovirus sequences, all belonging to type GI, were found in seven of the samples. Adenovirus and rotavirus hits were sporadically detected across all sampling sites and upon further investigation showed mainly adenovirus C and rotavirus A hits.

It is known that noroviruses, astroviruses and rotaviruses follow a winter seasonality and enteroviruses follows a summer seasonality pattern^38–40^. The time of sampling of the sewage was in a 3-month timeframe between January and March, which corresponds to the winter period in the northern hemisphere, therefore a higher prevalence of winter seasonal viruses was expected in those. When looking at the global distribution of viruses, the average abundance of astro- and noroviruses was higher in the northern hemisphere, and the reverse pattern was observed for enteroviruses, with higher average abundance in the southern hemisphere during the sampling period (Fig. 5c). Given the cross-sectional nature of our study we acknowledge that these seasonal patterns will have to be confirmed using longitudinal sampling which would allow for meaningful statistical analysis, but our first observations align with what is generally expected at that time of the year.

## Discussion

This global sewage study gives, for the first time, a catch-all metagenomic comparison of the urban sewage virome of major cities across the world. We show that it is possible to detect a wide diversity of viruses in sewage samples and we identify geographical and seasonal differences in abundance for specific viral groups, including those that are currently targeted by surveillance for diarrheal and neurological disease, as well as viruses that could be used as indicators for presence of specific mosquito species. In addition, we provide the global scientific community with a geographically very broad resource for searching for novel virus sequences as novel pathogens continue to emerge. The pilot study also highlights some important challenges that need to be addressed to take the technology forward, such as how to deal with low input samples and the overabundance of phages, plant, and insect viruses in the sample. Metagenomic sequencing of viruses is a complex and evolving technology which is currently far from being standardized. Differences in sample preprocessing, sequencing technology, and data analysis can have a major impact on the viral read abundance, diversity, and the proportion of sequences that are annotated^41,42^. In our study, we eliminated lab-to-lab variability by performing all sample preparation, sequencing and analysis at the same location, which, apart from the analysis, is obviously not feasible for global surveillance. Further work is ongoing, including the development of fieldable sample treatment and sequencing protocols, comparison of effects of sample preparation on viral richness and further exploration of applicability, by longitudinal sampling and sampling in the presence of known ongoing outbreaks.

A critical challenge of using metagenomic sequencing for surveillance purposes remains the interpretation of sequence annotations. With the development of high-speed k-mer based annotation tools such as the ones used in this study, annotation can be performed rapidly and with few false negatives. However, erroneous and mis-annotated entries in public databases, together with inconsistency in the sequence-based taxonomic classification of viruses, make annotation to the species level challenging. Major steps have been taken to create a more consistent sequence based viral taxonomy^27,43^, but these approaches have not yet been integrated in fast viral annotation tools. Also, deposits of large volumes of virus sequences without a clear host association or pathogenicity data in public databases^44^ make it difficult to interpret the relevance of such findings. In our data, many of these “environmental viruses” could be identified. Given the increase in virus diversity in reference databases, it is striking how many sequence reads can remain unclassified with the currently used methods. This is in line with previous observations, where 40–90% of the sequence reads could not be classified^45^. It can very well be that the currently unclassified sequence reads represent potential new viruses, including novel pathogens.

In conclusion, we show the potential of global viral surveillance using metagenomic sequencing of sewage without ignoring the complexity of the approach. However, with improvements in sample preprocessing, sequencing methods and interpretability of viral sequence annotation this potential will increase.

## Methods

### Urban sewage sample and metadata collection

Samples were obtained from 62 countries from all continents as previously described^26^. All samples were taken before wastewater treatment. A questionnaire was filled in with information on sampling site, sample consistency and sample temperature, including transport time, storage time, and temperature before shipping. All samples were taken in a timeframe of 3 months from January until March 2016. In addition to sample specific data, additional metadata (Supp. File 1) was collected such as demographics, type of industry in the surrounding area, weather conditions and catchment area of the sewer. Upon arrival, samples were thawed at room temperature and 250 ml of the raw sewage was taken and centrifuged at 10,000 *g* for 10 min. The pellet was removed for bacterial content determination and DNA metagenomic sequencing^26^ and the supernatant was used to perform the virus specific sample pretreatment and sequencing.

### Sample processing for sequencing

Viral extraction was performed on 40 ml of sewage supernatant as previously described^46^. In short, the conductivity was measured to exceed 2000 µs and the pH of the samples was adjusted to pH 4. Afterwards 10 ml PEG 6,000 was added and the samples were incubated overnight at 4˚C under agitation.

After incubation the samples were centrifuged a 13,500 *g* for 1.5 h at 4 °C. The supernatant was removed, the pellet was dissolved in warm glycine buffer and 1 mL of chloroform-butanol (50/50) was added. After mixing, the sample was centrifuged for 5 min at 13,000 *g* at 4 °C. The filtrate was collected through a series of filters with 5 µm, 1.2 µm, 0.45 µm and 0.22 µm pore size.

Unprotected free DNA was removed by incubation with Ambion Turbo DNase for 30 min at 37 °C. Total nucleic acid content was extracted using Roche NA isolation kit and cDNA was made using superscript III (Invitrogen) using random hexamers that avoids amplification of human rRNA^47^. dsDNA was made using Klenow (NEB) and samples were sheared using Ion Shear Plus Enzyme Mix II. Libraries were amplified for 15 cycles using High Fidelity Platinum PCR reaction. The library concentration was determined using Ion Torrent quantification kit (Thermo Fisher). If the concentration was below 20 nM, extra amplification cycles were performed. Sequencing was performed on the Ion Torrent S5XL platform to generate around 10 million sequence reads per sample.

### Data preprocessing

Raw fastq files were quality trimmed using FastP^48^. Read ends were trimmed to mean quality 25 with a sliding window of 5. Reads were trimmed to 400 nucleotides by default because the chemistry of Ion Torrent sequencing technology allows for reads of maximally 400 nucleotides long and longer reads were observed to contain high Phred score non-sense repetitive patterns in the tail region. Reads shorter than 50 nucleotides were discarded as well as reads with an average Phred score below 25. Duplicate reads were removed using CD-HIT^49^ by clustering reads that start at the exact same position in the genome and have over 90% sequence identity in the first 50 nucleotides of the read, because of variable read length and observed insertion and deletion errors in the beginning of the reads.

### Read based analysis

Due to the expected high diversity of viruses present in the sewage samples, a read based annotation of the data was chosen, contrary to an assembly-based approach. Annotation was performed using two taxonomic annotation tools: Kaiju^50^ and Centrifuge^51^. Kaiju performs taxonomic annotation based on an amino acid (AA) level which provides a higher sensitivity. This is especially important for the annotation of viral sequences given the high mutation rate of viruses^52^ compared to other organisms. In parallel with Kaiju, Centrifuge was run, which uses nucleotide (nt) identity for taxonomic annotation. Combining a nucleotide and an amino acid based matching approach ensures that both coding and non-coding read sequences can be annotated. In addition, the combination of two read annotation tools with different annotation strategies was chosen to give more robust mapping results.

The databases used for taxonomic annotation consisted of archaeal, bacterial and human RefSeq sequences and were extended with all viral and phage entries in GenBank version 230^53^ because of the limited viral and phage sequence diversity in the RefSeq database.

Recommended quality thresholds and parameters for metagenomic data were used for both Kaiju and Centrifuge. Kaiju was run in greedy mode with a score cutoff of 70 and an error of 5. Centrifuge was run with a score threshold of 300 and a hit length cutoff of 50. If neither method produced a hit the read was annotated as “Unknown”. BASTA^54^ was used to determine the last common ancestor (LCA) of each hit given by both methods without restrictions on hit quality.

The final read counts passing QC were determined by the sum of read annotations at a certain taxonomic level and were normalized by total dereplicated read count to adjust for differences in sequencing depth and data quality^55–57^. The LCA taxon was used if the annotation at a certain taxonomic level was absent. Manual regrouping of taxonomic levels was performed to calculate read counts of human pathogenic viruses and read counts by host group. For sample comparison, read counts were normalized by Hellinger transformation^58^. Sample-wise comparison was done by calculating the Bray–Curtis dissimilarity between the normalized read counts using the R package Vegan^59^. Further investigation of the annotation of specific viral species was performed by mapping the reads against a redundant set of reference genomes using KMA with default parameters^60^. The maps of global read distribution were created using the continent subdivision from the “rnaturalearthdata” R package and the Köppen-Geiger climate classification^61^.

### Supplementary information

Supplementary file1

## Data Availability

Raw sequence data that support the findings of this study have been deposited in the European Nucleotide Archive with the study accession code PRJEB23496.

## Appendix

See Extended Data Table 1.

**Table 1:** List of viral families and viral species detected in other metagenomic sewage surveillance studies.

Virus family	Virus species	References
Adenoviridae	Human adenovirus B	^20^
	Human adenovirus C	^20^
	Human adenovirus F7 201–332	^20^
	Human adenovirus 41	^19^
Astroviridae	Human astrovirus 1	^18,19^
	Human astrovirus 3	^18^
	Human astrovirus 4	^18^
	Human astrovirus 8	^18^
	Astrovirus MLB1	^19^
Caliciviridae	Norwalk virus	^18,19^
	Sapporo virus	^18,19^
Hepeviridae	Hepatitis E virus	^18^
Papillomaviridae	Human papillomavirus 112	^19^
	Papillomaviridae	^20^
Parvoviridae	Adeno-associated virus	^18,19^
	Human bocavirus 2	^18,19^
	Human bocavirus 3	^18,19^
Picobirnaviridae	Human picobirnavirus	^18,19^
Picornaviridae	Human Enterovirus B	^20^
	Aichi virus	^18,19^
	Human cosavirus D	^18^
	Human coxsackievirus B2	^18^
	Human coxsackievirus B6	^18^
	Human echovirus 11	^18^
	Human enterovirus 76	^18^
	Human enterovirus 97	^18^
	Human parechovirus 1	^18^
	Human poliovirus 2	^18^
	Saffold virus	^18^
	Salivirus NG-J1	^18^
	Human klassevirus 1/Salivirus NG-J1	^19^
	Human parechovirus 1	^19^
	Human parechovirus 3	^19^
	Human parechovirus 4	^19^
	Human parechovirus 7	^19^
Polyomaviridae	JC polyomavirus	^20^
	BK polyomavirus	^20^
	Polyomavirus HPyV6	^19^
Reoviridae	Banna virus	^18^

## References

[1] Gomes MFC (2014). Assessing the international spreading risk associated with the 2014 west african ebola outbreak. PLoS Curr..

[2] Koopmans M (2019). Familiar barriers still unresolved—a perspective on the Zika virus outbreak research response. Lancet Infect. Dis..

[3] Thézé J (2018). Genomic epidemiology reconstructs the introduction and spread of Zika virus in Central America and Mexico. Cell Host Microbe.

[4] Glennon EE, Jephcott FL, Restif O, Wood JLN (2019). Estimating undetected Ebola spillovers. PLoS Negl. Trop. Dis..

[5] Peeling RW, Murtagh M, Olliaro PL (2019). Epidemic preparedness: Why is there a need to accelerate the development of diagnostics?. Lancet Infect. Dis..

[6] Nieuwenhuijse DF, Koopmans MPG (2017). Metagenomic sequencing for surveillance of food- and waterborne viral diseases. Front. Microbiol..

[7] Chan EH (2010). Global capacity for emerging infectious disease detection. Proc. Natl. Acad. Sci..

[8] World Health Organization. A research and development Blueprint for action to prevent epidemics. (2019). Available at: https://www.who.int/blueprint/en/.

[9] Grubaugh ND (2019). Tracking virus outbreaks in the twenty-first century. Nat. Microbiol..

[10] Aarestrup FM (2012). Integrating genome-based informatics to modernize global disease monitoring, information sharing, and response. Emerg. Infect. Dis..

[11] Holmes EC, Rambaut A, Andersen KG (2018). Pandemics: Spend on surveillance, not prediction. Nature.

[12] Gardy JL, Loman NJ (2018). Towards a genomics-informed, real-time, global pathogen surveillance system. Nat. Rev. Genet..

[13] Neiderud CJ (2015). How urbanization affects the epidemiology of emerging infectious diseases. Afr. J. Disabil..

[14] Callender DM (2018). Factors contributing to and strategies to combat emerging arboviruses. Global Public Health.

[15] Van Der Avoort HGAM, Reimerink JHJ, Ras A, Mulders MN, Van Loon AM (1995). Isolation of epidemic poliovirus from sewage during the 1992–3 type 3 outbreak in the Netherlands. Epidemiol. Infect..

[16] Asghar H (2014). Environmental surveillance for polioviruses in the global polio eradication initiative. J. Infect. Dis..

[17] Kaliner E (2015). The Israeli public health response to wild poliovirus importation. Lancet Infect. Dis..

[18] Ng TFF (2012). High variety of known and new RNA and DNA viruses of diverse origins in untreated sewage. J. Virol..

[19] Cantalupo PG (2011). Raw sewage harbors diverse viral populations. MBio.

[20] Aw TG, Howe A, Rose JB (2014). Metagenomic approaches for direct and cell culture evaluation of the virological quality of wastewater. J. Virol. Methods.

[21] Niedrig M, Patel P, El Wahed AA, Schädler R, Yactayo S (2018). Find the right sample: A study on the versatility of saliva and urine samples for the diagnosis of emerging viruses. BMC Infect. Dis..

[22] Gourinat A-C, O’Connor O, Calvez E, Goarant C, Dupont-Rouzeyrol M (2015). Detection of Zika virus in urine. Emerg. Infect. Dis..

[23] Benschop KSM (2017). Polio and measles down the drain: Environmental enterovirus surveillance in the Netherlands, 2005 to 2015. Appl. Environ. Microbiol..

[24] Drosten C (2013). Clinical features and virological analysis of a case of Middle East respiratory syndrome coronavirus infection. Lancet. Infect. Dis..

[25] Wang X-W (2005). Concentration and detection of SARS coronavirus in sewage from Xiao Tang Shan Hospital and the 309th Hospital. J. Virol. Methods.

[26] Hendriksen RS (2019). Global monitoring of antimicrobial resistance based on metagenomics analyses of urban sewage. Nat. Commun..

[27] Bolduc B (2017). vConTACT: An iVirus tool to classify double-stranded DNA viruses that infect Archaea and Bacteria. PeerJ.

[28] McMinn BR, Ashbolt NJ, Korajkic A (2017). Bacteriophages as indicators of faecal pollution and enteric virus removal. Lett. Appl. Microbiol..

[29] Calero-Cáceres W, Balcázar JL (2019). Antibiotic resistance genes in bacteriophages from diverse marine habitats. Sci. Total Environ..

[30] Rosario K, Symonds EM, Sinigalliano C, Stewart J, Breitbart M (2009). Pepper mild mottle virus as an indicator of fecal pollution. Appl. Environ. Microbiol..

[31] Tijssen P, Pénzes JJ, Yu Q, Pham HT, Bergoin M (2016). Diversity of small, single-stranded DNA viruses of invertebrates and their chaotic evolutionary past. J. Invertebr. Pathol..

[32] Boonnak K, Suttitheptumrong A, Jotekratok U, Pattanakitsakul SN (2015). Phylogenetic analysis reveals genetic variations of densovirus isolated from field mosquitoes in bangkok and surrounding regions. Southeast Asian J. Trop. Med. Public Health.

[33] Ng TFF (2011). Broad surveys of DNA viral diversity obtained through viral metagenomics of mosquitoes. PLoS ONE.

[34] Palinski R (2017). A novel porcine circovirus distantly related to known circoviruses is associated with porcine dermatitis and nephropathy syndrome and reproductive failure. J. Virol..

[35] Vu D-L, Cordey S, Brito F, Kaiser L (2016). Novel human astroviruses: Novel human diseases?. J. Clin. Virol..

[36] Kroneman A (2011). An automated genotyping tool for enteroviruses and noroviruses. J. Clin. Virol..

[37] van Beek J (2018). Molecular surveillance of norovirus, 2005–16: An epidemiological analysis of data collected from the NoroNet network. Lancet Infect. Dis..

[38] Ahmed SM, Lopman BA, Levy K (2013). A systematic review and meta-analysis of the global seasonality of norovirus. PLoS ONE.

[39] Thongprachum A, Khamrin P, Maneekarn N, Hayakawa S, Ushijima H (2016). Epidemiology of gastroenteritis viruses in Japan: Prevalence, seasonality, and outbreak. J. Med. Virol..

[40] Pons-Salort M (2018). The seasonality of nonpolio enteroviruses in the United States: Patterns and drivers. Proc. Natl. Acad. Sci. USA..

[41] Smits SL (2015). Recovering full-length viral genomes from metagenomes. Front. Microbiol..

[42] Hjelms MH (2017). Evaluation of methods for the concentration and extraction of viruses from sewage in the context of metagenomic sequencing. PLoS ONE.

[43] Aiewsakun P, Simmonds P (2018). The genomic underpinnings of eukaryotic virus taxonomy: Creating a sequence-based framework for family-level virus classification. Microbiome.

[44] Shi M (2016). Redefining the invertebrate RNA virosphere. Nature.

[45] Krishnamurthy SR, Wang D (2017). Origins and challenges of viral dark matter. Virus Res..

[46] Schaeffer J (2018). Improving the efficacy of sewage treatment decreases norovirus contamination in oysters. Int. J. Food Microbiol..

[47] Endoh D (2005). Species-independent detection of RNA virus by representational difference analysis using non-ribosomal hexanucleotides for reverse transcription. Nucleic Acids Res..

[48] Chen S, Zhou Y, Chen Y, Gu J (2018). Fastp: An ultra-fast all-in-one FASTQ preprocessor. Bioinformatics.

[49] Fu L, Niu B, Zhu Z, Wu S, Li W (2012). CD-HIT: Accelerated for clustering the next-generation sequencing data. Bioinformatics.

[50] Menzel P (2016). Fast and sensitive taxonomic classification for metagenomics with Kaiju. Nat. Commun..

[51] Kim D, Song L, Breitwieser FP, Salzberg SL (2016). Centrifuge: Rapid and sensitive classification of metagenomic sequences. Genome Res..

[52] Duffy S, Shackelton LA, Holmes EC (2008). Rates of evolutionary change in viruses: Patterns and determinants. Nat. Rev. Genet..

[53] Benson DA (2012). GenBank. Nucleic Acids Res..

[54] Kahlke T, Ralph PJ (2019). BASTA—Taxonomic classification of sequences and sequence bins using last common ancestor estimations. Methods Ecol. Evol..

[55] Solonenko SA (2013). Sequencing platform and library preparation choices impact viral metagenomes. BMC Genom..

[56] Gomez-Alvarez V, Teal TK, Schmidt TM (2009). Systematic artifacts in metagenomes from complex microbial communities. ISME J..

[57] Schmieder R, Edwards R (2011). Fast identification and removal of sequence contamination from genomic and metagenomic datasets. PLoS ONE.

[58] Legendre P, Gallagher ED (2001). Ecologically meaningful transformations for ordination of species data. Oecologia.

[59] Oksanen, J. *et al.* vegan: Community Ecology Package. (2019).

[60] Clausen PTLC, Aarestrup FM, Lund O (2018). Rapid and precise alignment of raw reads against redundant databases with KMA. BMC Bioinform..

[61] Rubel F, Kottek M (2010). Observed and projected climate shifts 1901–2100 depicted by world maps of the Köppen–Geiger climate classification. Meteorol. Z..

