# Author Correction: Setting a baseline for global urban virome surveillance in sewage

**DOI:** 10.1038/s41598-021-95934-3

**Published:** 2021-08-25

**Authors:** David F. Nieuwenhuijse, Bas B. Oude Munnink, My V. T. Phan, the Global Sewage Surveillance project consortium, Rene S. Hendriksen, Artan Bego, Catherine Rees, Elizabeth Heather Neilson, Kris Coventry, Peter Collignon, Franz Allerberger, Teddie O. Rahube, Guilherme Oliveira, Ivan Ivanov, Thet Sopheak, Yith Vuthy, Christopher K. Yost, Djim-adjim Tabo, Sara Cuadros-Orellana, Changwen Ke, Huanying Zheng, Li Baisheng, Xiaoyang Jiao, Pilar Donado-Godoy, Kalpy Julien Coulibaly, Jasna Hrenovic, Matijana Jergović, Renáta Karpíšková, Bodil Elsborg, Mengistu Legesse, Tadesse Eguale, Annamari Heikinheimo, Jose Eduardo Villacis, Bakary Sanneh, Lile Malania, Andreas Nitsche, Annika Brinkmann, Courage Kosi Setsoafia Saba, Bela Kocsis, Norbert Solymosi, Thorunn R. Thorsteinsdottir, Abdulla Mohamed Hatha, Masoud Alebouyeh, Dearbhaile Morris, Louise O’Connor, Martin Cormican, Jacob Moran-Gilad, Antonio Battisti, Patricia Alba, Zeinegul Shakenova, Ciira Kiiyukia, Eric Ng’eno, Lul Raka, Aivars Bērziņš, Jeļena Avsejenko, Vadims Bartkevics, Christian Penny, Heraa Rajandas, Sivachandran Parimannan, Malcolm Vella Haber, Pushkar Pal, Heike Schmitt, Mark van Passel, Milou G.M. van de Schans, Tina Zuidema, Gert-Jan Jeunen, Neil Gemmell, Kayode Fashae, Astrid Louise Wester, Rune Holmstad, Rumina Hasan, Sadia Shakoor, Maria Luz Zamudio Rojas, Dariusz Wasyl, Golubinka Bosevska, Mihail Kochubovski, Cojocaru Radu, Amy Gassama†, Vladimir Radosavljevic, Moon Y.F. Tay, Rogelio Zuniga-Montanez, Stefan Wuertz, Dagmar Gavačová, Marija Trkov, Karen Keddy, Kerneels Esterhuyse, Marta Cerdà-Cuéllar, Sujatha Pathirage, D.G.Joakim Larsson, Leif Norrgren, Stefan Örn, Tanja Van der Heijden, Happiness Houka Kumburu, Ana Maria de RodaHusman, Berthe-Marie Njanpop-Lafourcade, Pawou Bidjada, Somtinda Christelle Nikiema-Pessinaba, Belkis Levent, John Scott Meschke, Nicola Koren Beck, Chinh Van Dang, Doan Minh Nguyen Tran, Nguyen Do Phuc, Geoffrey Kwenda, Patrick Munk, Shweta Venkatakrishnan, Frank M. Aarestrup, Matthew Cotten, Marion P. G. Koopmans

**Affiliations:** 1 Viroscience Department, Erasmus Medical Center, Rotterdam, The Netherlands; 2 National Food Institute, Technical University of Denmark, Lyngby, Denmark; 3 Institute of Public Health, Tirana, Albania; 4 Melbourne Water Corporation, Docklands, Australia; 5 University of Copenhagen, Frederiksberg C, Australia; 6 Applied Research, Docklands, Australia; 7 Canberra Hospital, Canberra, Australia; 8 Austrian Agency for Health and Food Safety (AGES), Vienna, Austria; 9 Botswana International University of Science and Technology, Palapye, Botswana; 10 Vale Institute of Technology, Sustainable Development, Belém, Brazil; 11 National Center of Infectious and Parasitic Diseases, Sofia, Bulgaria; 12 Institut Pasteur du Cambodge, Phnom Penh, Cambodia; 13 University of Regina, Regina, Canada; 14 University of N’Djamena, N’Djamena, Chad; 15 Centro de Biotecnología de los Recursos Naturales, Universidad Católica del Maule, Talca, Chile; 16 Guangdong Provincial Center for Disease Control and Prevention, Guangzhou, China; 17 Shantou University Medical College, Shantou, China; 18 Corporacion Colombiana de Investigacion Agropecuaria (AGROSAVIA), Mosquera, Colombia; 19 Institut Pasteur de Côte d’Ivoire, Abidjan, Côte d’Ivoire; 20 Faculty of Science, University of Zagreb, Zagreb, Croatia; 21 Andrija Stampar Teaching Institute of Public Health, Zagreb, Croatia; 22 Veterinary Research Institute, Brno, Czech Republic; 23 Renseanlæg Lynetten, København K, Denmark; 24 Addis Ababa University, Addis Ababa, Ethiopia; 25 University of Helsinki, Helsinki, Finland; 26 Instituto Nacional de Investigación en Salud Pública-INSPI (CRNRAM), Quito, Galápagos, Ecuador; 27 National Public Health Laboratories, Ministry of Health and Social Welfare, Kotu Layout, Kotu, Gambia; 28 National Center for Disease Control and Public Health, Tbilisi, Georgia; 29 Robert Koch Institute, Berlin, Germany; 30 University for Development Studies, Tamale, Ghana; 31 Semmelweis University, Institute of Medical Microbiology, Budapest, Hungary; 32 University of Veterinary Medicine, Budapest, Hungary; 33 Institute for Experimental Pathology, University of Iceland, Keldur, Reykjavík, Iceland; 34 Cochin University of Science and Technology, Cochin, India; 35 Pediatric Infections Research Center, Research Institute for Children’s Health, Shahid Beheshti University of Medical Sciences, Tehran, Iran; 36 National University of Ireland Galway, Galway, Ireland; 37 School of Public Health, Ben Gurion University of the Negev and Ministry of Health, Beer-Sheva, Israel; 38 Istituto Zooprofilattico Sperimentale del Lazio e della Toscana, Rome, Italy; 39 National Center of Expertise, Taldykorgan, Kazakhstan; 40 Mount Kenya University, Thika, Kenya; 41 Kenya Medical Research Institute, Nairobi, Kenya; 42 University of Prishtina “Hasan Prishtina” & National Institute of Public Health of Kosovo, Pristina, Kosovo; 43 Institute of Food Safety, Animal Health and Environment “BIOR”, Riga, Latvia; 44 Institute of Food Safety, Riga, Latvia; 45 Luxembourg Institute of Science and Technology, Belvaux, Luxembourg; 46 Centre of Excellence for Omics-Driven Computational Biodiscovery, Faculty of Applied Sciences, AIMST University, Kedah, Malaysia; 47 Environmental Health Directorate, St. Venera, Malta; 48 Agriculture and Forestry University, Kathmandu, Nepal; 49 National Institute for Public, Health and the Environment (RIVM), Bilthoven, Netherlands; 50 Wageningen Food Safety Research, Wageningen, Netherlands; 51 University of Otago, Dunedin, New Zealand; 52 University of Ibadan, Ibadan, Nigeria; 53 Norwegian Institute of Public Health, Oslo, Norway; 54 VEAS, Slemmestad, Norway; 55 Aga Khan University, Karachi, Pakistan; 56 National Institute of Health, Lima, Peru; 57 National Veterinary Research Institute, Puławy, Poland; 58 Institute of Public Health of the Republic of Macedonia, Skopje, Republic of Macedonia; 59 State Medical and Pharmaceutical University, Chişinău, Republic of Moldova; 60 Institut Pasteur de Dakar, Dakar, Sénégal; 61 Institute of Veterinary Medicine of Serbia, Belgrade, Serbia; 62 Nanyang Technological University Food Technology Centre (NAFTEC), Nanyang Technological University (NTU), Singapore, Singapore; 63 Nanyang Technological University, Singapore Centre for Environmental Life Sciences Engineering (SCELSE), Singapore, Singapore; 64 Public Health Authority of the Slovak Republic, Bratislava, Slovakia; 65 National Laboratory of Health, Environment and Food, Ljubljana, Slovenia; 66 University of the Witwatersrand, Johannesburg, South Africa; 67 Daspoort Waste Water Treatment Works, Pretoria, South Africa; 68 IRTA, Centre de Recerca en Sanitat Animal (CReSA, IRTA-UAB), Bellaterra, Spain; 69 Medical Research Institute, Colombo, Sri Lanka; 70 The Sahlgrenska Academy at the University of Gothenburg, Gothenburg, Sweden; 71 Swedish University of Agricultural Sciences, Uppsala, Sweden; 72 Ara region bern ag, Herrenschwanden, Switzerland; 73 Kilimanjaro Clinical Research Institute, Moshi, Tanzania; 74 Centre for Infectious Disease Control, Bilthoven, the Netherlands; 75 Agence de Médecine Préventive, Dapaong, Togo; 76 National Institute of Hygiene, Lome, Togo; 77 Division of Integrated Surveillance of Health Emergencies and Response, Lomé, Togo; 78 Public Health Institution of Turkey, Ankara, Turkey; 79 University of Washington, Seattle, USA; 80 Institute of Public Health in Ho Chi Minh City, Ho Chi Minh, Viet Nam; 81 University of Zambia, Lusaka, Zambia

Correction to: *Scientific Reports* 10.1038/s41598-020-69869-0, published online 13 August 2020

The Acknowledgements section in the original version of this Article was incomplete.

“This study has received funding from the European Union’s Horizon 2020 research and innovation program under Grant agreement no. 643476 (COMPARE), the World Health Organization, and The Novo Nordisk Foundation (NNF16OC0021856: Global Surveillance of Antimicrobial Resistance). We would like to thank Miranda de Graaf for the technical assistance at Erasmus MC.”

now reads:

“This study has received funding from the European Union’s Horizon 2020 research and innovation program under Grant agreement no. 643476 (COMPARE), the World Health Organization, and The Novo Nordisk Foundation (NNF16OC0021856: Global Surveillance of Antimicrobial Resistance). My V. T. Phan was supported by a Marie Sklodowska-Curie Individual Fellowship, funded by the EU H2020 research and innovation programme (Grant Agreement No. 799417). We would like to thank Miranda de Graaf for the technical assistance at Erasmus MC.”

The original Article has been corrected.

